# Professional competency framework for occupational health nurses in Brazil

**DOI:** 10.1590/0034-7167-2024-0592

**Published:** 2025-11-28

**Authors:** Daniela Sarreta Ignacio, Laura Andrian Leal, Iasmin Gabrielli Silva, Denise Maria Osugui, Fabiana Faleiros, Silvia Helena Henriques

**Affiliations:** I Faculdade Dr. Francisco Maeda. Ituverava, São Paulo, Brazil; II Universidade de São Paulo. Ribeirão Preto, São Paulo, Brazil

**Keywords:** Occupational Health Nursing, Professional Competence, Staff Development, Nurse’s Role, Professional Training., Enfermería del Trabajo, Competencia Profesional, Desarrollo del Personal, Rol de la Enfermera, Capacitación Profesional.

## Abstract

**Objectives:**

to develop a framework of core competencies for occupational health nurses based on their professional experience.

**Methods:**

an exploratory qualitative study, carried out with occupational health nurses linked to Brazilian and multinational companies, with data collection from January to May 2021, through an electronic form (sociodemographic data), followed by a semi-structured interview. Inductive thematic data analysis was carried out. The study was approved by the Research Ethics Committee.

**Results:**

eight core professional competencies for occupational health nurses were identified: legal; effective communication; staff and service management; relational; improvement of work processes; continuing education; health and safety; and research. Their knowledge and skills were synthesized in a competency framework.

**Final Considerations:**

the construction of a framework for occupational health nurses should guide managers and training centers in improving these professionals’ knowledge and skills within organizations and academia, aiming at changing their profile and professional recognition.

## RESUMEN

**Objetivos:**

desarrollar una matriz de competencias esenciales para enfermeras/os de salud ocupacional, basada en su experiencia profesional.

**Métodos:**

estudio cualitativo exploratorio, realizado con enfermeras/os de salud ocupacional que trabajan en empresas brasileñas y multinacionales. La recopilación de datos se realizó de enero a mayo de 2021 mediante un formulario electrónico (datos sociodemográficos), seguido de una entrevista semiestructurada. Se realizó un análisis temático inductivo de los datos. El estudio fue aprobado por el Comité de Ética de la Investigación.

**Resultados:**

se identificaron ocho competencias profesionales esenciales para las enfermeras de salud ocupacional: legal; comunicación efectiva; gestión de equipos y servicios; relacional; mejora de los procesos de trabajo; formación continua; salud y seguridad; e investigación. Sus conocimientos y habilidades se resumieron en una matriz de competencias.

**Consideraciones Finales:**

la construcción de la matriz para enfermeros del trabajo debe orientar a los gestores y centros de formación en la mejora de los conocimientos y habilidades de estos profesionales dentro de las organizaciones y la academia, visando la modificación de su perfil y reconocimiento profesional.

## INTRODUCTION

One of the areas in which nurses are involved is occupational nursing, which focuses on preventing diseases and maintaining worker health. Occupational health nurses (OHNs) are present in industries, companies and other types of organizations, where they perform functions related to care, surveillance, education, research, management, and auditing^(1)^, which requires these professionals to master specific skills for their work.

Professional competency is understood as the use of behaviors that guarantee deliveries/production and results at work resulting from the integrated use of knowledge, skills, and attitudes in association with good professional performance^(2)^.

The demand for professional competencies directed at OHNs by the job market also leads institutions to broaden their organizational vision. By recognizing this professional’s potential, added to that of the work team, they are consolidated as a corporate differential, towards excellence in the competitive market by preserving productive force integrity and health^(3)^.

In this regard, mapping competencies relevant to the different levels of training allows unveiling the performance profiles inherent to care practice, providing support to compare, for instance, educational training profiles (higher and specialized) with job market demands^(4)^.

In the international scenario, there are several studies that have identified and validated the core competencies for OHNs, such as communication, health and safety promotion, risk management, service improvement, self-development, ethics and legislation, health education, and occupational health^(5-10)^.

In the national literature, there are studies that describe OHNs’ actions and attributions, identify isolated competencies, without presenting how they are fully constituted^(11,12)^, or only provide a summary of attributions, responsibilities or activities^(13,14)^. This data is an integral part of the competencies; however, it disregards their composition by knowledge and skills necessary for OHNs’ performance.

In this study, the theoretical frameworks considered, with regard to the term “professional competency”, were the French school, which understands them as the set of knowledge, skills and attitudes^(15)^, and the Royal College of Nursing (RCN) Competency Framework model for specific competencies for OHNs, which presents them with a distinction between core professional competencies (communication, health, safety and security, personal and people management, service improvement, quality, and equality and diversity) and those specialized for OHNs.

The Competency Framework developed by the RCN describes four levels of complexity in a progression system (from beginner to expert), which gradually expands the responsibilities and the exercise of new skills by OHNs, being used in assessments and career progression as it has clear criteria for analyzing professionals^(16)^. It is known that the RCN is not adapted to the Brazilian context, but there is no evidence in Brazil of a framework of competencies specific to OHNs available in literature.

That said, it is believed that, based on the identification of core competencies by OHNs, with the construction of the Brazilian competency framework, it will be possible for the Specialized Service in Safety Engineering and Occupational Medicine (In Portuguese, *Serviço Especializado em Engenharia de Segurança e Medicina do Trabalho* - SEESMT) team and in organizations to recognize its role, contributing to appreciation. Given the above, this study presents the following guiding questions: which competencies are essential for OHNs? What knowledge and skills constitute them?

This study aims to develop a professional competency framework for Brazilian OHNs based on their professional reality. The complexity of the Brazilian context, characterized by a diversity of economic sectors, social inequality and challenges in health and safety policies at work, requires specialized and continuous training of these professionals, ensuring that they are prepared to act effectively and in an integrated manner.

By answering the study’s questions, it is also proposed to provoke reflection among national training centers in the reorganization of their pedagogical projects, adapting disciplines to OHNs’ work practice. Scientifically, the recognition of OHNs’ work and the unveiling of their professional reality are expected, which is intended in the emergence of studies in this reality, considering the vision, experience and construction of occupational nursing according to its actors.

## OBJECTIVES

To develop a framework of professional skills for Brazilian OHNs based on their professional experience.

## METHODS

### Ethical aspects

The study was approved by the Research Ethics Committee, official letter 267/2020 dated 09/09/2020. Participants signed the Informed Consent Form, in accordance with Resolution 466/2012 of the Brazilian National Health Council^(17)^. Anonymity was guaranteed by using the acronym OHN (occupational health nurse) in the statements, followed by the order in which the interview was conducted (e.g., OHN 1; OHN 34).

The data obtained will be archived for five years (2021-2026), and access will be authorized exclusively for the responsible researchers and by the Research Ethics Committee of the proposing institution, if requested. After this period, all records will be deleted, in order to avoid any type of identification of participants.

### Study design

This is an exploratory study with a qualitative approach to data. The COnsolidated criteria for REporting Qualitative research checklist, recommended by the Equator Network^(18)^, was used to present the article for its use in validating research reports that collect and analyze data through interviews.

### Study setting

The study setting consisted of national or multinational companies, headquartered in Brazil, urban and rural, with risk level III or IV, in which SEESMT has a linked OHN (n=34), as established by the New Regulatory Standard 04^(19)^. They were companies with a single OHN, responsible for the head office and all its branches, even if located in different Brazilian states.

### Data source

Eligible participants were nurses specialized in occupational health nursing from all over the country, of both sexes, with at least one year of professional experience as an OHN and inserted in the job market in the area. Workers who were away from work for more than three months, due to sick leave or maternity leave, were excluded.

The snowball technique was used together with virtual social networks (VSN) to recruit and invite professionals and effectively collect data^(20)^, with the research being publicized on several VSN such as Facebook^®^, Instagram^®^, LinkedIn^®^, and in specific nurses’ groups on WhatsApp^®^. The first participant originated from the sending of an invitation letter in a WhatsApp^®^ group of graduate students from a Brazilian public higher education institution, with the access link to an electronic form characterizing OHNs and the company in which they work.

### Data collection

Data collection took place from January to May 2021 in two stages: electronic form for collecting sociodemographic data; and semi-structured interviews, via video call, due to the SARS-CoV-2 pandemic.

Data collection was carried out by the main author, a master’s degree holder and professor at a university in the countryside of São Paulo, with extensive experience and training in data collection techniques. Before the interviews began, the study objectives and relevance for academic and professional practice were presented. The interview script (validated by professors with a doctoral degree in the Southeast region (n=10)) contained the following questions: a) Which professional competencies do you identify as essential for OHNs? b) What knowledge is necessary for this competency? C) What skills do you perform/use within this competency? The interviews were individual, with a maximum duration of 60 minutes, with audio recording and transcribed by the main researcher. Data collection was concluded when theoretical data saturation was reached^(21)^.

The interviews took place remotely at the time established by participants. The number of participants and the number of interviews conducted were determined based on the point at which the power of information was reached, with data collection ending when the study objective was achieved^(22)^. Transcription was done manually, without the use of software, to familiarize participants with the data. Finally, it is worth noting that the data were disclosed to participants after the study was completed, and the article was also published.

### Data analysis

The inductive thematic analysis method^(23)^ was used to extract data from the interviews, going through the following stages: data transcription and reading; systematic coding of interesting data characteristics; research of topics grouping codes; review and verification of the topics to which coding extraction should respond; analysis to refine the details of each topic; and final reanalysis of selected excerpts from the responses to the research guidance questions, in order to answer them.

It is worth noting that the analysis was carried out by the main researcher, combined with the careful review and analysis validity by the other authors of this study, which contributed to the process of reflectiveness of the findings.

## RESULTS

### Participant characterization

The final sample consisted of 34 professionals, 27 (89.2%) of whom were female, 12 (35.5%) aged 31 to 35 years old and with an average age of 40 years old, 29 (85.29%) with more than ten years of professional experience in nursing, and 15 (44.12%) with more than five years as OHNs. The distribution, according to the region of the country in which OHNs practice their profession, shows a greater concentration in the Southeast region (86%), followed by the South and Central-West regions (n=2), with 5% each, and the others with 3% (n=1). It should be noted that there was only one OHN per company, and the same person is responsible for the head office and branches throughout the country, regardless of the productive activity, operating in up to five different states (n=25 OHNs).

It was observed that 14 (36.84%) of OHNs reported specializations aimed at the development of managerial competencies, distributed in the area of outpatient management, public or hospital health management.

### Core professional skills for occupational health nurses

**Figure 1:**
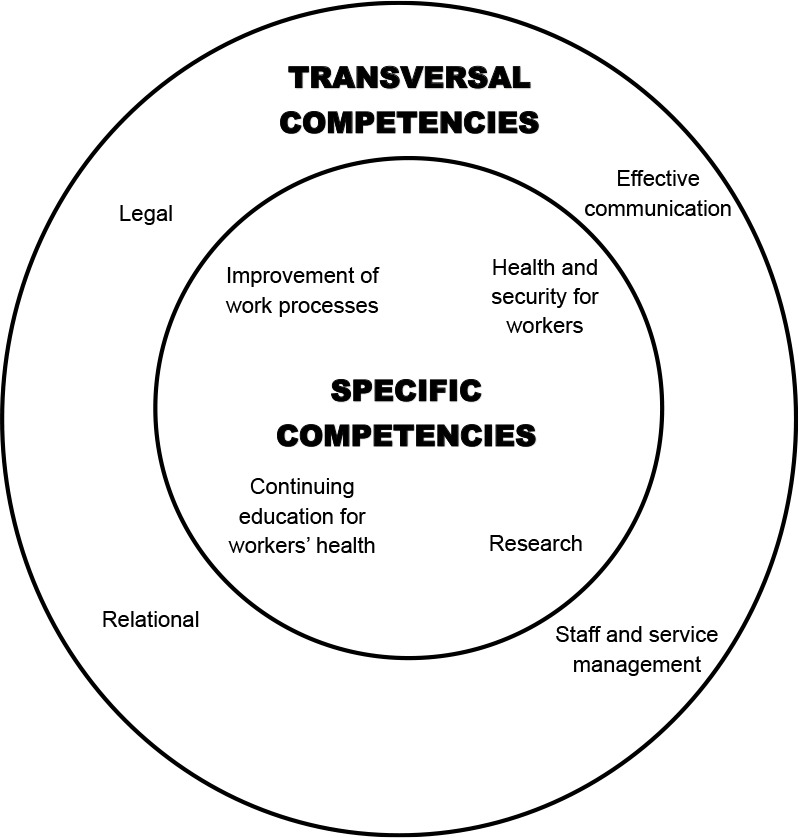
Illustrative schematic model of core professional competencies of Brazilian occupational health nurses according to their professional practice (N=34), Ribeirão Preto, São Paulo, Brazil, 2024

From the speeches, eight core professional competencies to OHN emerged, which were distributed into two categories of competencies: transversal (effective communication, legal, and staff and service management), as they permeate all other competencies, and specific (improvement of work processes, health and security for workers, continuing education for workers’ health, and research), represented in Figure 1. It is noteworthy that, for each competency, knowledge and skills related to them were identified.

### Legal

Legal competency presupposes the need for knowledge related to labor legislation, such as the Consolidation of Labor Laws, regulation of self-employed professionals, existing forms of labor contracts, among others.

[...] *it is necessary to master legislation, the legislative issues of our activities. We have to comply with what comes from the federal, state and municipal spheres. These are laws that need to be known in order to apply the actions.* (OHN 07)

*You must be well aware of all the rules and procedures that must be followed, not only by the company, but also the rules and procedures established by regulatory bodies.* (OHN 26)

For this competency, skills such as clarity in communication, good interpersonal relationships, self-development, implementation of the Systematization of Nursing Care in Occupational Health, knowing how to work in a team and planning skills were identified.

*It is necessary to have the ability to interpret and apply legislation, to understand that there are regional characteristics that need to be known* [...] *equity to work with differences, providing the same standard of treatment, behavior to promote justice and social freedom, ability to plan internal health policies* [...] *combined with legal obligations.* (OHN 07)

### Staff and service management

The knowledge for this competency must emphasize the management of occupational risks, associated with knowledge of administration, statistics, communication and information technology, which are added to health management.

*Manage the team, manage the entire team work process, knowledge of the humanization and ethics of service, knowledge of what your company does and what the work process of this worker is.* (OHN 08)

As for OHN skills, these include delegating work activities, defining team activities, establishing partnerships, creating strategies, knowing how to communicate, having skills to manage programs and data, as well as cultivating relationships, as shown in the speech:

*Define activities, what each person will do,* [...] *delegate functions,* [...] *of our activities so that they run more smoothly* [...] *really be the most efficient and effective* [...] *effective communication and good relationships with everyone to correctly develop the tasks. That they are done in the best possible way and bring support to the company.* (OHN 22)

### Effective communication

For this competency, participants reported the need for knowledge of communication and expression in all its forms (formal and informal, verbal and non-verbal, graphics...), combined with mastery of information technology and foreign languages.

*It is the domain of technology, not only IT, but the issue of intranet, internet, various means of communication, and* [...] *all forms of communication that exist: verbal, written, formal communication, informal,* [...] *graphs, spreadsheets, tables, health indicators, because these are all ways of transmitting information,* [...] *I would add the body issue, which also has a great influence.* (OHN 24)

Furthermore, skills were associated with this competency, such as reading, interpreting and writing text, with the use of technological tools (software, internet and applications) that must be added to the skills of having mastery of other languages as well as Brazilian Sign Language.

*I would call tables, maps, and graphs graphic communication. The numerical part of occupational health indicators also falls within communication.* (OHN 13)

### Relational

Large areas of knowledge were identified by OHNs to guarantee the performance of relational competency, as they call it, encompassing ethics, mastery of the vernacular language for its expression in formal and informal ways, written or spoken, didactics, intermediate to advanced knowledge in IT, among other knowledge, as shown in the dialogues.

[...] *in order to relate well with others, a person should necessarily have more than one language, intermediate to advanced knowledge of Word and Excel, knowledge of computer programs that we have today, to optimize our work.* (OHN 23)

For this competency, skills such as being able to value and welcome one’s own coworkers, planning intersectoral actions, developing assertive communication, working with diversity, among others, stood out.

[...] *knowing how to relate to people at different levels and in different situations* [...] *you have to know how to talk and understand, have a human relationship, a relationship with people with special needs, a relationship with people in high positions. People like attention, to be heard. This relationship, a good relationship, is part of the nursing profile for workers.* (OHN 17)

### Improvement of work processes

The knowledge required for OHNs for this competency involves mastery of legislation pertinent to the economic-industrial sector, knowledge of the company’s internal standards, auditing, among other knowledge required for OHNs’ professional practice.

[...] *it would be having knowledge of everything that exists within the company, knowing all the procedures that the company has, managing the procedures to be able to adequately disclose not only to the people around you, professionals, but also to staff.* (OHN 24)

Concerning the skills for this competency, participants highlighted being a leader, having communication skills, multidisciplinary teamwork, interprofessional relationships, networking, organization, autonomy, time management, people management, among others.

[...] *it’s as if we were an influencer, because leaders have this characteristic: they don’t determine what to do, but they can influence things to happen the way they should, or serve as an example, which is a very strong condition there, together with leadership there too.* (OHN 12)

### Continuing education for workers’ health

For continuing education, knowledge focused on the contents of foreign language proficiency, communication and expression, teaching and didactic strategies, legislation, and health education were highlighted.

*Yes, in terms of teaching, public speaking and classroom management, preparation, planning, courses, training and a series of other events that may take place and that have as their focus worker improvement.* (OHN 30)

*To have educational competency, you must have knowledge of research, investment in courses and training for your self-development.* (OHN 19)

As for skills, the most important were mastery of information technologies, use of different means to communicate and provide health guidance.

*The ability to give training, know how to speak in public, know the terms to use. Sometimes, you’re going to give a training and you have to know how to use a term that connects the worker to the director. Sometimes, we end up explaining a little, you have to be there, always with this ability.* (OHN 21)

### Health and security for workers

For this competency, the data revealed the need for knowledge in pharmacology, clinical medicine, emergency, communication and expression, among others.

*You have to know a lot, the most basic aspects of worker health: hearing health, musculoskeletal problems and ergonomic issues.* (OHN 19)

All this knowledge must be applied through skills such as interprofessional relationships, establishing partnerships, planning, decision-making, leadership, quality of work, and knowing how to forecast and provide materials.

*You need to know about worker health, teamwork, continuing education* [...] *really demonstrate your competency, in technical and theoretical terms, demonstrate safety. This is passed on to your team, and your team passes it on to the rest of the company* [...] *so that you can really develop the objectives proposed by the occupational health and safety team.* (OHN 11)

### Research

For the competency related to research, the mastery of knowledge such as epidemiology and health surveillance, biostatistics and statistics, research methods and techniques (epidemiological survey, impact study, document analysis, literature review, among others), expression and English was indicated.

[...] *in addition to knowing, you have to seek scientific basis* [...] *to be able to develop excellent work, you have to show the client why you are choosing that method. You have to have scientific basis.* (OHN 19)

[...] *knowing how to collect data, knowing how to inform, knowing how to analyze, using the appropriate tools to be able to transmit the information you have obtained.* (OHN 24)

In relation to skills, the need to know how to process and analyze data, to have mastery of ways of recording information and defining health indicators, planning skills, decision-making, organization and time management, among others, was identified.

*I see it as the identification of indicators, of correlations between a certain number of accidents or absences. It is a condition inherent to the position or inherent to the function they are performing, or to the environment in which they are working at that moment.* (OHN 32)

Based on the data identified, a competency framework proposal was constructed for OHNs, presented below in two charts: Chart 1, transversal core professional competencies for OHNs; and Chart 2, specific core competencies identified by these professionals with their knowledge and skills, according to their daily work and related to their entire work practice.

**Chart 1:** Transversal core competency framework for Brazilian occupational health nurses, Ribeirão Preto, São Paulo, Brazil, 2024

TRANSVERSAL COMPETENCIES	KNOWLEDGE	SKILLS
**LEGAL**	Social security legislation, worker health surveillance and e-Social; Labor regulatory standards; Labor legislation, ordinances, decrees and the case law that regulates it; Health legislation (Collegiate Board Resolution); Laws creating and regulating the Brazilian Health System.	Have clear communication; Perform collaborative work; Seek self-development; Carry out and execute planning; Have good interpersonal relationships; Ability to interpret and apply laws.
**STAFF AND SERVICE MANAGEMENT**	Legislation and ethics; Administration; Communication and expression; Information technology; Health management; Health surveillance; Statistics.	Know how to plan and delegate activities; Define team activities; Establish partnerships and teamwork; Develop leadership skills; Work with computers and technology; Know how to communicate; Create action strategies.
**EFFECTIVE COMMUNICATION**	Communication and expression; Languages ​​(Portuguese, English and French); Information and Communication Technologies; Software; Brazilian Sign Language.	Express themselves verbally and in writing; Synthesize and interpret texts, standards and data; Have command of the Office^®^ package; Communicate in another language(s); Process data and communicate visually/graphically; Be welcoming; Develop good human/interpersonal relationships; Encourage teamwork.
**RELATIONAL**	Communication and expression; Languages ​​(Portuguese, English and French); Ethics; Information and Communication Technologies; Didactics and teaching strategies; Brazilian Sign Language; Psychology.	Welcome people, ideas and knowing how to negotiate; Have the ability to interact and integrate; Make/follow the plan; Develop collective work; Maintain an assertive communication standard: Use emotional intelligence; Manage people; Work with diversity.

**Chart 2:** Specific core competency framework for Brazilian occupational health nurses, Ribeirão Preto, São Paulo, Brazil, 2024

SPECIFIC COMPETENCIES	KNOWLEDGE	SKILLS
**IMPROVEMENT OF WORK PROCESSES**	Knowledge of legislation relevant to the economic sector; Occupational health and safety; Knowledge of the company’s internal standards; Auditing and its verification systems (International Organization for Standardization, National Accreditation Organization and Joint Commission International); Information technology and software applied to the area; Ethics and deontology; Process and quality management.	Lead the work team with a view to people management and self-development; Have good communication skills and establish networking; Work in a multidisciplinary and team manner; Build interprofessional relationships (establish partnerships and intersectoral work); Plan, organize, manage time and develop autonomy (strategic business management); Build and apply quality indicators and tools; Adapt work processes.
**CONTINUING EDUCATION FOR WORKERS’ HEALTH**	Languages ​​(Portuguese, English and French); Didactics and teaching strategies; Legislation; Health education; Health topics; Information technology; Research.	Apply varied teaching strategies to transmit knowledge; Use the means of communication by employing Information and Communication Technologies and providing feedback; Synthesize the content; Act in a systematic manner, with planning and organization; Develop partnerships and work together, as a team and across sectors; Seek knowledge and self-development.
**HEALTH AND SECURITY FOR WORKERS**	Pharmacology and clinical medicine; Emergency; Communication and expression and teaching; Information and Communication Technologies; Legislation and auditing; Prevention and health promotion with strategic planning and risk management; Workers’ health (mental health, ergonomics, hygiene and safety at work, occupational diseases and health surveillance).	Carry out/adapt planning and make decisions; Lead and have autonomy; Seek quality in work and self-development; Predict and provide materials and supplies; Develop educational actions; Have argumentative skills; Keep records and write communications; Master technical and assistance skills; Supervise biosafety practices.
**RESEARCH**	Epidemiology (biostatistics) and health surveillance; Research methods and techniques; Auditing; Evidence-based nursing; Communication and expression; English language and computer science.	Collect, process and analyze data; Search for knowledge/study to offer/have technical-scientific support; Define health indicators; Master written communication, information recording systems and graphics; Have planning and decision-making, organization and time management skills; Develop partnerships.

## DISCUSSION

Data analysis made it possible to identify OHNs’ core competencies according to their professional practice and to propose a competency framework, considered by the literature as essential to the professions^(24,25)^, in which the organization of knowledge and skills in the form of a competency framework allows identifying the minimum competencies that are necessary and, at the same time, characterize the professional activity^(26)^.

The model adopted as a theoretical framework for analysis and construction of the competency framework proposal lists six core competencies whose skills are presented in increasing levels of complexity, according to the level of career progression^(16)^, which does not apply to the reality experienced by OHNs in Brazilian industry.

Among the identified competencies, four are of a transversal nature, called legal, effective communication, relational, and staff and service management. Transversal competencies present knowledge and skills that permeate the other competencies, and have the following characteristics: multifunctionality (necessary in different facets of life); transferability (used in the world of work, in personal life and in society, in career development); are based on cognition (constructed with active individual reflections, through critical thinking); multidimensionality (composed of different clusters of competencies, such as cognitive, interpersonal, business, preparation for work); are learned (continuous process through different contexts) and comprehensive (they are broad, involve explanations and the application of knowledge)^(27)^.

The development and applicability of transversal competencies at work depend on the ability to articulate knowledge in response to changes at the organizational, sectoral and institutional levels, integrating content (theory and practice), which requires their improvement in the face of changes in the work context^(28)^.

When dealing with legal competency, researchers state that, as it is related to ethics and legislation, it requires knowledge, application and respect for the laws that order and guide any type or modality of work performed or not by this professional^(29)^, which reinforces its characteristics of essentiality and transversality^(30)^, proven by identifying its core knowledge in five of the reported competencies, demonstrating that this competency is fundamental in ensuring that OHNs’ practices are aligned with legal and ethical requirements, when acting to provide a safe and healthy work environment for all workers.

For competency staff and service management, there are studies that highlight the need for knowledge in statistics, Systematization of Nursing Care in Occupational Health, information technology, strategic planning, legislation (health, labor, social security and social security), people management and teamwork^(8,9,31)^, supporting the results identified in the study.

It is important to note that management is one of the activities exclusive to nurses, regardless of their work context. The importance of in-service management includes the implementation of management models that allow planning, deciding, organizing and controlling the provision of care, through the use of management practices aimed at greater effectiveness and efficiency in the application of resources^(32)^, which, for OHNs, become a daily challenge, in the search for better working conditions and quality of services offered to staff of an organization. Thus, their action must be based on instruments, means and competencies.

Communication is a core competency that intersects with management, identified in actions related to decision-making, in the use of social skills, visible in team management, in the sharing of updated information with management in the strategic and financial areas^(33)^. By using communication, OHNs establish an interpersonal relationship with the team, a fact that is relevant to professionals’ performance and the organization’s success. Therefore, there is relevance to the knowledge and skills related to relational competency, which intersects with effective communication.

In the group of other core competencies, participants highlighted the need to be fluent in more than one language, whether English or French, because some companies are multinational and workers need to communicate with employers and workers who do not speak Portuguese. Nurses’ ability to interact productively with people from different cultural backgrounds is necessary to provide greater quality and results in services^(34,35)^, in addition to enabling them to learn about other strategies and work realities.

Reflection on improving the work process as an occupational health professional competency translates into the implementation of practices that increase the efficiency, safety and quality of occupational health services. Occupational health professionals must be able to identify areas for improvement, develop effective strategies and lead change initiatives that benefit both workers and the organization^(35,36)^.

It is known that OHNs are responsible for identifying and assessing possible risks in the work environment. Therefore, he is required to implement prevention and control measures, in addition to guiding staff on safety standards. Thus, having the competency for health and safety at work is relevant in daily work, a fact supported by scientific literature^(9,37)^.

Competency research has several knowledge and skills that have been identified in other competencies, which reinforces its importance and applicability^(38,39)^.

All competencies require specific knowledge and skills, but it is important to point out that this knowledge is intertwined in the various competencies presented. Moreover, there are recurring skills, such as interpersonal relationships, which are reflected in developing partnerships and collaborative work, teamwork, and networking. This skill is described as fundamental in professional practice due to interaction with the multidisciplinary team^(34)^.

Hence, for these competencies to be incorporated into practice, strategies can be implemented by the organization or by the workers themselves. Managers must encourage continuous learning for the development of their staff, going beyond traditional models that aim to fill gaps in knowledge or specific skills in professional practice^(40)^.

New research should be conducted on professional competencies for other categories in the area of occupational health, such as occupational nursing technicians.

### Study limitations

This study presents as limitations of access to professionals its dispersed distribution in Brazil and the SARS-CoV-2 pandemic, expanding OHNs’ activities in companies (assistance and management), which restricted their availability to participate in the study.

### Contributions to nursing, health or public policy

The construction of a competency framework for OHNs should guide managers and provoke reflection in national training centers in the reorganization of their pedagogical projects, adapting the disciplines to OHNs’ work practice, by improving these professionals’ knowledge and skills and the academic area, aiming at changing their profile, professional recognition and appreciation. Scientifically, the recognition of OHNs’ work is expected, by revealing their professional reality, in the emergence of studies in this reality, considering the vision, experience and construction of occupational nursing according to its actors.

## FINAL CONSIDERATIONS

Core competencies to their professional practice were identified through OHNs’ perception, and based on these, a professional competency framework was constructed. This framework can assertively direct OHN training according to the needs of the position and its functions, by recognizing their competencies and identifying the gaps in the training of this professional.

This framework allows for a better visualization of the knowledge and skills of each competency, which should favor its recognition within organizations, with the division of paradigms regarding its inestimable value to worker health, elevating the profession to a new level.

It is urgent to invest in research that investigates OHNs’ practice in Brazil, assisting health care bodies and systems in investing in worker health and strengthening the area that deals with maintaining the economically active population’s health in the country.

## Data Availability

The research data are available only upon request.
