# Supplementary material for: Professional competency framework for occupational health nurses in Brazil
Source: Rev Bras Enferm. 2025 Nov 28;78(5):e20240592. doi: 10.1590/0034-7167-2024-0592 (PMC12673613; doi:10.1590/0034-7167-2024-0592)
Supplement: Translation

# Translation: Matriz de competências profissionais do enfermeiro do trabalho para o Brasil

**DOI:** 10.1590/0034-7167-2024-0592pt

**Authors:** Daniela Sarreta Ignacio, Laura Andrian Leal, Iasmin Gabrielli Silva, Denise Maria Osugui, Fabiana Faleiros, Silvia Helena Henriques

**Affiliations:** I Faculdade Dr. Francisco Maeda. Ituverava, São Paulo, Brasil; II Universidade de São Paulo. Ribeirão Preto, São Paulo, Brasil

**Keywords:** Enfermeiro do Trabalho, Competências Profissionais, Desenvolvimento de Pessoal, Papel do Profissional de Enfermagem, Capacitação Profissional.

## Abstract

**Objetivos:**

desenvolver uma matriz de competências essenciais do enfermeiro do trabalho a partir de sua experiência profissional.

**Métodos:**

estudo qualitativo exploratório, realizado com enfermeiros do trabalho vinculados a empresas brasileiras e multinacionais, com a coleta de dados de janeiro a maio de 2021, por formulário eletrônico (dados sociodemográficos), seguido por entrevista semiestruturada. Realizou-se a análise temática indutiva dos dados. O estudo foi aprovado pelo Comitê de Ética em Pesquisa.

**Resultados:**

identificaram-se oito competências profissionais essenciais ao enfermeiro do trabalho: jurídica; comunicação eficaz; gerência da equipe e do serviço; relacional; aprimoramento dos processos de trabalho; educação permanente; saúde e segurança; e pesquisa. Seus conhecimentos e habilidades foram sintetizados em uma matriz de competências.

**Considerações Finais:**

a construção da matriz para o enfermeiro do trabalho deve orientar gestores e centros formadores no aprimoramento dos conhecimentos e habilidades desses profissionais junto às organizações e área acadêmica, visando mudança de perfil e reconhecimento profissional.

## INTRODUÇÃO

Uma das áreas em que o enfermeiro se insere é a enfermagem do trabalho, voltada à prevenção de doenças e manutenção da saúde dos trabalhadores. O enfermeiro do trabalho (ET) está presente em indústrias, empresas e outros tipos de organizações, onde desempenha funções relacionadas à assistência, vigilância, educação, pesquisa, gestão e auditoria^(1)^, o que exige desses profissionais o domínio de competências específicas para a sua atuação.

Entende-se por competência profissional o emprego de comportamentos que garantam entregas/produção e resultados no trabalho decorrentes do uso integrado de conhecimentos, habilidades e atitudes em associação para o bom desempenho profissional^(2)^.

A exigência de competências profissionais direcionada ao ET pelo mercado de trabalho também conduz as instituições à ampliação de sua visão organizacional. Ao reconhecer as potencialidades desse profissional, somadas às da equipe de trabalho, consolidam-se como um diferencial corporativo, rumo à excelência no mercado competitivo pelo preservar da integralidade e da saúde da força produtiva^(3)^.

Nessa direção, o mapeamento das competências pertinentes aos diferentes níveis de formação permite o desvelar dos perfis de atuação inerentes à prática assistencial, trazendo subsídios para confrontar, por exemplo, perfis de formação educacional (superior e especializada) com as exigências do mercado de trabalho^(4)^.

No cenário internacional, há vários estudos que identificaram e validaram as competências essenciais ao ET, tais como comunicação, promoção de saúde e segurança, gestão de riscos, melhoria do serviço, autodesenvolvimento, ética e legislação, educação em saúde, e saúde ocupacional^(5-10)^.

Na literatura nacional, há estudos que descrevem as ações e atribuições do ET, identificam competências isoladas, sem apresentar como são integralmente constituídas^(11,12)^, ou trazem apenas um sumário de atribuições, responsabilidades ou atividades^(13,14)^. Esses dados são parte integrante das competências, entretanto desconsideram sua composição por saberes e habilidades necessários ao desempenho do ET.

Neste estudo, consideraram-se como referenciais teóricos, no que diz respeito ao termo “competência profissional”, a escola francesa, que as compreende como o conjunto de conhecimentos e habilidades e atitudes^(15)^, e o modelo de Matriz de Competências do *Royal College of Nursing* (RCN) para as competência específicas ao ET, o qual as apresenta com distinção entre as competências profissionais essenciais (comunicação, saúde segurança e proteção, gestão de pessoas e de pessoal, melhoria do serviço, qualidade, e igualdade e diversidade) e as especializadas para o ET.

A Matriz de Competências elaboradas pelo RCN descreve quatro níveis de complexidade em um sistema de progressão (de inicial a *expert*), que amplia gradativamente as suas responsabilidades e o exercício de novas habilidades pelo ET, sendo empregue nas avaliações e progressão na carreira por possuir critérios claros de análise do profissional^(16)^. Sabe-se que o RCN não está adaptado ao contexto brasileiro, mas não há evidências no Brasil de matriz de competências específicas ao ET disponibilizadas na literatura.

Isto posto, acredita-se que, a partir da identificação das competências essenciais pelo próprio ET, com a construção da matriz de competências brasileira, será possível o reconhecimento de seu papel pela equipe do Serviço Especializado em Engenharia de Segurança e Medicina do Trabalho (SEESMT) e nas organizações, contribuindo para sua valorização. Diante do exposto, este estudo apresenta as seguintes questões norteadoras: quais competências são essenciais para o ET? Quais são os conhecimentos e habilidades que as constituem?

Este estudo tem como objetivo desenvolver uma de matriz de competências profissionais para o ET brasileiro com base em sua realidade profissional. A complexidade do contexto brasileiro, caracterizada por uma diversidade de setores econômicos, desigualdade social e desafios em políticas de saúde e segurança no trabalho, exige uma formação especializada e contínua desses profissionais, garantindo que estejam preparados para atuar de forma eficaz e integrada.

Ao responder aos questionamentos do estudo, também propõe-se provocar a reflexão dos centros formadores nacionais na reorganização de seus projetos pedagógicos, adequando as disciplinas à práxis trabalhadora do ET. Cientificamente, esperam-se o reconhecimento do fazer do ET e o desvelar de sua realidade profissional, o que se pretende no despontar de estudos nesta realidade, considerando a visão, a experiência e a construção da enfermagem do trabalho conforme seus atores.

## OBJETIVOS

Desenvolver uma de matriz de competências profissionais para o ET brasileiro a partir de sua experiência profissional.

## MÉTODOS

### Aspectos éticos

O estudo foi aprovado pelo Comitê de Ética em Pesquisa com Seres Humanos, ofício nº 267/2020 de 09/09/2020. Os participantes assinaram o Termo de Consentimento Livre e Esclarecido, conforme Resolução n^o^ 466/2012 do Conselho Nacional de Saúde^(17)^. O anonimato foi garantido pela utilização da sigla ET (enfermeiro ocupacional) nas falas, seguida da ordem em que a entrevista foi realizada (*e.g*., ET 1; ET 34).

Os dados obtidos foram arquivados por cinco anos (2021-2026), e o acesso será autorizado exclusivamente para os pesquisadores responsáveis e pelo Comitê de Ética em Pesquisa da instituição proponente, caso seja solicitado. Após este período, todos os registros serão apagados, de modo a evitar qualquer tipo de identificação dos participantes.

### Tipo de estudo

Estudo exploratório, de abordagem qualitativa dos dados. Utilizou-se para a apresentação do artigo a lista de verificação *COnsolidated criteria for REporting Qualitative research*, recomendado pela Rede Equator^(18)^, por seu emprego na validação de relatos de pesquisa que coletam e analisam dados por meio de entrevistas.

### Cenário do estudo

O cenário do estudo constituiu-se por empresas nacionais ou multinacionais, sediadas no Brasil, urbanas e rurais, com grau de risco III ou IV, nas quais o SEESMT possui ET vinculado (n=34), conforme estabelece a Nova Norma Regulamentadora nº 04^(19)^. Eram empresas com único ET, responsável pela matriz e todas as suas filiais, mesmo que localizadas em estados brasileiros diferentes.

### Fonte de dados

Os participantes elegíveis foram enfermeiros especialistas em enfermagem do trabalho de todo o território nacional, de ambos os sexos, com no mínimo um ano de experiência profissional como ET e inseridos no mercado de trabalho na área. Os trabalhadores em afastamento do serviço por tempo superior a três meses, por motivo de licença saúde ou licença maternidade, foram excluídos.

Empregou-se a técnica bola de neve junto à ferramenta das redes sociais virtuais (RSV) para a captação e convite dos profissionais e efetiva coleta de dados^(20)^, com a divulgação da pesquisa em várias RSV como *Facebook* ^®^, *Instagram* ^®^, *LinkedIn* ^®^, e em grupos específicos de enfermeiros no WhatsApp^®^. O primeiro participante se originou do disparo da carta convite em um grupo de WhatsApp^®^de alunos de pós-graduação de uma instituição de ensino superior pública brasileira, com o *link* de acesso a um formulário eletrônico de caracterização do ET e da empresa em que trabalha.

### Coleta de dados

A coleta de dados ocorreu no período de janeiro a maio de 2021 em duas etapas: formulário eletrônico para a coleta de dados sociodemográficos; e entrevistas semiestruturadas, por videochamada, em decorrência da pandemia de SARS-CoV-2.

A coleta de dados foi realizada pela autora principal, mestre e docente de uma universidade do interior de São Paulo, com ampla experiência e treinamento na técnica de coleta de dados. Antes do início das entrevistas, foram realizadas apresentação dos objetivos e relevância do estudo para prática acadêmica e profissional. O roteiro de entrevistas (validado por professores doutores na área da região Sudeste (n=10)) continha as seguintes questões: a) Quais competências profissionais você identifica como essenciais para o ET? b) Quais os conhecimentos necessários para esta competência? C) Quais são as habilidades que você executa/utiliza dentro desta competência? As entrevistas foram individuais, com duração máxima de 60 minutos, com gravação de áudio e transcritas pela pesquisadora principal. A coleta de dados foi encerrada ao alcançar a saturação teórica dos dados^(21)^.

As entrevistas ocorreram de forma remota, no horário estabelecido pelos participantes. O número de participantes e a quantidade de entrevistas realizadas foram determinados com base no ponto em que o poder da informação foi alcançado, encerrando-se a coleta de dados ao atingir o objetivo do estudo^(22)^. A transcrição se deu de forma manual, sem o emprego de *software*, para aproximação e familiarização com os dados. Por fim, ressalta-se que os dados foram divulgados aos participantes após a conclusão do estudo, com disponibilização também da publicação do artigo.

### Análise de dados

Utilizou-se o método análise temática indutiva^(23)^ para extração dos dados das entrevistas, ao percorrer as etapas: transcrição e leitura dos dados; codificação sistemática de características interessantes dos dados; pesquisa de tópicos agrupando códigos; revisão e verificação dos tópicos aos quais a extração da codificação deve responder; análise para refinar os detalhes de cada tópico; e reanálise final de trechos selecionados das respostas às questões de orientação de pesquisa, a fim de respondê-las.

Destaca-se que a análise foi realizada pela pesquisadora principal, aliada à cuidadosa revisão e validação da análise pelos demais autores deste estudo, o que contribuiu para o processo de reflexividade dos achados.

## RESULTADOS

### Caracterização dos participantes

A amostra final foi composta por 34 profissionais, sendo 27 (89,2%) do sexo feminino, 12 (35,5%) na faixa etária de 31 a 35 anos e média de idade de 40 anos, 29 (85,29%) com mais de dez anos de experiência profissional na enfermagem, e 15 (44,12%) com mais de cinco anos como ET. A distribuição, segundo a região do país em que os ETs exercem sua profissão, mostra maior concentração na região Sudeste (86%), seguida da região Sul e Centro-Oeste (n=2), com 5% cada, e as demais com 3% (n=1). Salienta-se que era apenas um ET por empresa, e o mesmo responde pela matriz e filiais existentes em todo o país, independentemente da atividade produtiva, atuando em até cinco estados diferentes (n=25 ETs).

Observou-se que 14 (36,84%) dos ETs relataram especializações direcionadas ao desenvolvimento de competências gerenciais, distribuídas na área de gestão ambulatorial, gestão em saúde pública ou hospitalar.

### Competências profissionais essenciais ao enfermeiro do trabalho

A partir dos discursos, emergiram oito competências profissionais essenciais ao ET, que foram distribuídas em duas categorias de competências: as transversais (comunicação eficaz, jurídica, e gerência da equipe e do serviço), por perpassarem todas as demais competências, e as específicas (aprimoramento dos processos de trabalho, saúde e segurança para o trabalhador, educação permanente à saúde do trabalhador, e pesquisa), representada na Figura 1. Ressalta-se que, para cada competência, identificaram-se os conhecimentos e as habilidades relativos a elas.

**Figura 1:** Modelo esquemático ilustrativo das competências profissionais essenciais ao enfermeiro do trabalho brasileiro segundo sua práxis profissional (N=34), Ribeirão Preto, São Paulo, Brasil, 2024

### Jurídica

A competência jurídica pressupõe a necessidade de saberes relacionados à legislação trabalhista, a exemplo da Consolidação das Leis do Trabalho, regulamentação dos profissionais autônomos, formas de contrato trabalhistas existentes, entre outros.

[...] *é preciso dominar a legislação, as questões legislativas de nossas atividades. Nós temos que atender o que vem das esferas federal, estadual e municipal. São legislações que precisam ser conhecidas para aplicação das ações.* (ET 07)

*Tem que estar bem ciente de todas as normas e procedimentos que tem de ser seguidos, não só pela empresa, mas como normas e procedimentos estabelecidos pelos órgãos reguladores.* (ET 26)

Para esta competência, identificaram-se habilidades como clareza na comunicação, boa relação interpessoal, autodesenvolvimento, implementação da Sistematização da Assistência de Enfermagem em Saúde Ocupacional, saber trabalhar em equipe e habilidades de planejamento.

*É necessário a habilidade de interpretação e da aplicabilidade da legislação, entender que existem características regionais que precisam ser conhecidas* [...] *equidade para trabalhar as diferenças dando mesmo padrão de tratamento, de comportamento para promover a justiça e a liberdade social, habilidade para planejar políticas de saúde internas* [...] *casado com as obrigações legais.* (ET 07)

### Gerência da equipe e do serviço

Os conhecimentos para esta competência devem enfatizar a gestão dos riscos ocupacionais, associados ao conhecimento de administração, estatística, comunicação e informática, que se somam à gestão em saúde.

*Administrar a equipe, administrar todo o processo de trabalho da equipe, conhecimento da parte de humanização e ética no atendimento, conhecimento do que sua empresa faz e de qual é o processo de trabalho deste trabalhador.* (ET 08)

Quanto às habilidades do ET, incluem-se delegar as atividades de trabalho, definir as atividades da equipe, estabelecer parcerias, criar estratégias, saber se comunicar, ter habilidades para gerenciar programas e dados, além de cultivar relacionamentos, como mostra o discurso:

*Definir atividades, o que cada um vai fazer,* [...] *delegar funções,* [...] *das nossas atividades para que elas decorram com mais facilidade* [...] *realmente ser o mais eficiente e eficaz* [...] *uma comunicação efetiva e bom relacionamento com todos para desenvolver corretamente as tarefas. Que elas sejam feitas da melhor maneira possível e tragam respaldo para empresa.* (ET 22)

### Comunicação eficaz

Para esta competência, os participantes relataram a necessidade de conhecimentos de comunicação e expressão em todas as suas modalidades (formal e informal, verbal e não verbal, gráficos...), aliados ao domínio de tecnologia da informação e línguas estrangeiras.

*É o domínio de tecnologia, não só informática, mas a questão da intranet, internet, meios de comunicação variados, e* [...] *todas as formas de comunicação que se tem: verbal, escrita, a comunicação formal, a informal,* [...] *gráficos, planilhas, tabelas, indicadores de saúde, porque isso tudo são formas de transmitir informações,* [...] *eu acrescentaria a questão corporal, que também influencia bastante.* (ET 24)

Além disso, habilidades foram associadas a essa competência, tais como leitura, interpretação e escrita de texto, com a utilização de ferramentas tecnológicas (*softwares*, internet e aplicativos) que devem ser acrescidas às habilidades de ter o domínio de outros idiomas, como também de Língua Brasileira de Sinais.

*Eu chamaria de comunicação gráfica tabelas, mapas, gráficos. A parte numérica dos indicadores em saúde ocupacional também entra dentro da comunicação.* (ET 13)

### Relacional

Grandes áreas de conhecimento foram identificadas pelos ETs para a garantia do desempenho da competência relacional, assim denominada por eles, englobando a ética, o domínio da língua vernácula para sua expressão de modo formal e informal, escrito ou falado, a didática, o conhecimento intermediário a avançado em informática, entre outros saberes, como mostram diálogos.

[...] *a pessoa deveria, para se relacionar bem com os outros, obrigatoriamente ter uma língua a mais, um conhecimento de intermediário a avançado em Word e Excel, conhecimento em programas de computação que tem hoje, para otimizar o nosso trabalho.* (ET 23)

Para tal competência, destacaram-se habilidades como ser capaz de valorizar e acolher os próprios colegas de trabalho, planejar ações intersetoriais, desenvolver comunicação assertiva, trabalhar com as diversidades, entre outras.

[...] *saber se relacionar com pessoas dos mais diferentes níveis e situações* [...] *tem que saber conversar e entender, ter uma relação humana, relação com pessoas com necessidades especiais, relação com pessoas de alto escalão. As pessoas gostam de atenção, de ser ouvidas. Esse relacionamento, um bom relacionamento, faz parte do perfil da enfermagem para o trabalhador.* (ET 17)

### Aprimoramento dos processos de trabalho

Os conhecimentos necessários para o ET para esta competência envolvem domínio da legislação pertinente ao setor econômico-industrial, ciência das normas internas da empresa, auditoria, entre outros saberes, exigidos para o exercício profissional do ET.

[...] *seria ter conhecimento de tudo que existe dentro da empresa, saber todos os procedimentos que a empresa tem, gerenciar os procedimentos para poder divulgar adequadamente não só para as pessoas que estão ao seu redor, de profissionais, como para os colaboradores também.* (ET 24)

Quanto às habilidades para essa competência, os participantes destacaram ser líder, ter capacidade de comunicação, trabalho multidisciplinar em equipe, relacionamento interprofissional, *networking*, organização, autonomia, gestão do tempo, gestão de pessoas, entre outras.

[...] *é como se a gente fosse um influenciador, porque o líder tem essa característica: ele não determina que se faça, mas ele consegue influenciar para que as coisas aconteçam da forma com que deveriam, ou servir de exemplo, que é uma condição muito forte ali junto da liderança aí também.* (ET 12)

### Educação permanente à saúde do trabalhador

Para a educação permanente, apontaram-se conhecimentos voltados aos conteúdos de domínio de línguas estrangeiras, comunicação e expressão, estratégias de ensino e didática, legislação, e educação em saúde.

*Sim, na questão da didática, da oratória e do domínio de sala, da preparação, do planejamento, de cursos, capacitações e uma série de outros eventos que possam acontecer e que tenham como vertente o aprimoramento do trabalhador.* (ET 30)

*Para ter competência educacional, tem que ter conhecimento de pesquisa mesmo, investimento em cursos e treinamentos no seu autodesenvolvimento.* (ET 19)

Quanto às habilidades, destacaram-se o domínio de tecnologias de informação, utilização de diversos meios para se comunicar e realizar orientações em saúde.

*Habilidade de dar treinamentos, saber falar em público, saber os termos que se utiliza. Às vezes, você vai dar um treinamento e tem que saber usar um termo que una do peão ao diretor. Às vezes, a gente acaba explicando um pouco, tem que estar ali, sempre com essa habilidade.* (ET 21)

### Saúde e segurança para o trabalhador

Para esta competência, os dados revelaram necessidade de conhecimentos nas áreas da farmacologia, clínica médica, urgência e emergência, comunicação e expressão, entre outras.

*Tem que saber muito, saúde do trabalhador da parte mais básica: saúde auditiva, problemas osteomusculares e questões de ergonomia.* (ET 19)

Todo esse conhecimento deve ser aplicado por meio de habilidades como relacionamento interprofissional, estabelecimento de parcerias, planejamento, tomada de decisão, liderança, qualidade do trabalho, e saber realizar a previsão e provisão de materiais.

*É preciso saber sobre saúde do trabalhador, trabalho em equipe, educação permanente* [...] *realmente mostrar a sua competência, em termos técnicos e teóricos, mostrar segurança. Isso passa para sua equipe, e a sua equipe passa para o restante da empresa* [...] *para que você consiga realmente desenvolver os objetivos propostos pela equipe de saúde e segurança do trabalho.* (ET 11)

### Pesquisa

Para a competência relacionada à pesquisa, foi apontado o domínio de conhecimentos como epidemiologia e vigilância em saúde, bioestatística e estatística, métodos e técnicas de pesquisa (levantamento epidemiológico, estudo de impacto, análise documental, revisão de literatura, entre outros), expressão e inglês.

[...] *além de conhecer, tem que buscar embasamento científico* [...] *para poder desenvolver um trabalho com excelência, você tem que mostrar para o cliente o porquê está escolhendo aquela forma. Tem que ter embasamento científico.* (ET 19)

[...] *saber coletar os dados, saber informar, saber analisar, utilizar as ferramentas adequadas para poder transmitir o que você obteve de informações.* (ET 24)

A respeito das habilidades, identificaram-se necessidade de saber tratar e analisar dados, ter domínio dos modos de registro das informações e definição de indicadores de saúde, habilidades de planejamento, tomada de decisão, organização e gestão do tempo, entre outras.

*Eu vejo como a identificação dos indicadores, das correlações entre um determinado número de acidentes ou afastamentos. É uma condição inerente ao cargo ou inerente à função que ele está desempenhando, ou ao ambiente em que ele está trabalhando naquele momento.* (ET 32)

A partir dos dados identificados, foi construída uma proposta de matriz de competências para o ET, apresentada, na sequência, em dois quadros: Quadro 1, as competências profissionais essenciais transversais do ET; e Quadro 2, as competências essenciais específicas identificadas por esses profissionais com seus conhecimentos e habilidades, conforme o seu cotidiano laboral e relacionadas a toda sua práxis trabalhadora.

**Quadro 1:** Matriz de competências essenciais transversais para o enfermeiro do trabalho brasileiro, Ribeirão Preto, São Paulo, Brasil, 2024

COMPETÊNCIAS TRANSVERSAIS	CONHECIMENTOS	HABILIDADES
**JURÍDICA**	Legislação previdenciária, vigilância em saúde do trabalhador e e-Social; Normas regulamentadoras do trabalho ; Legislação trabalhista, portarias, decretos e as jurisprudências que a regulamentam; Legislação sanitária (Resolução da Diretoria Colegiada); Leis de criação e regulamentação do Sistema Único de Saúde.	Ter clareza na comunicação; Realizar trabalho colaborativo; Buscar seu autodesenvolvimento; Realizar e executar o planejamento; Ter bom relacionamento interpessoal; Capacidade de interpretar e aplicar as leis.
**GERÊNCIA DA EQUIPE E DO SERVIÇO**	Legislação e ética; Administração; Comunicação e expressão; Informática; Gestão em saúde; Vigilância em saúde; Estatística.	Saber planejar e delegar atividades; Definir as atividades de equipe; Estabelecer parcerias e trabalho em equipe; Desenvolver a liderança/liderar; Trabalhar com informática e tecnologia; Saber se comunicar; Criar estratégias de ação.
**COMUNICAÇÃO EFICAZ**	Comunicação e expressão; Idiomas (português, inglês e francês); Tecnologias de Informação e Comunicação; *Softwares*; Língua Brasileira de Sinais.	Expressar-se de modo verbal e escrito; Sintetizar, interpretar textos, normas e dados; Ter domínio do pacote *Office®*; Comunicar-se em outro(s) idioma(s); Fazer o tratamento de dados e comunicação visual/gráfica; Ter acolhimento; Desenvolver bom relacionamento humano/ interpessoal; Estimular o trabalho em equipe.
**RELACIONAL**	Comunicação e expressão; Idiomas (português, inglês e francês); Ética; Tecnologias de Informação e Comunicação; Didática e estratégias de ensino; Língua Brasileira de Sinais; Psicologia.	Acolher pessoas, ideias e saber negociar; Ter capacidade de interação e integração; Fazer/seguir o planejamento; Desenvolver o trabalho coletivo; Manter padrão de comunicação assertiva: Utilizar inteligência emocional; Realizar a gestão de pessoas; Trabalhar com as diversidades.

**Quadro 2:** Matriz de competências essenciais específicas para o enfermeiro do trabalho brasileiro, Ribeirão Preto, São Paulo, Brasil, 2024

COMPETÊNCIAS ESPECÍFICAS	CONHECIMENTOS	HABILIDADES
**APRIMORAMENTO DOS PROCESSOS DE TRABALHO**	Domínio da legislação pertinente ao setor econômico; Saúde e segurança no trabalho; Ciência das normas internas da empresa; Auditoria e os seus sistemas de verificação (*International Organization for Standardization*, Organização Nacional de Acreditação e *Joint Commission International*); Informática e *softwares* aplicados à área; Ética e deontologia; Gestão de processos e de qualidade.	Liderar a equipe de trabalho com vistas à gestão de pessoas e autodesenvolvimento; Ter boa capacidade de comunicação e estabelecer *networking*; Trabalhar de forma multidisciplinar e em equipe; Constituir relacionamento interprofissional (estabelecer parcerias e intersetorialidade); Planejar, organizar, gerir o tempo e desenvolver autonomia (gestão estratégica de negócios); Construir e aplicar indicadores e ferramentas de qualidade; Adaptar os processos de trabalho.
**EDUCAÇÃO PERMANENTE À SAÚDE DO TRABALHADOR**	Idiomas (português, inglês e francês); Didática e estratégias de ensino; Legislação; Educação em saúde; Tópicos de saúde; Informática; Pesquisa.	Aplicar estratégias de ensino variadas para transmitir o conhecimento; Utilizar os meios de comunicação ao empregar as Tecnologias de Informação e Comunicação e dar *feedbacks*; Sintetizar os conteúdos; Agir sistematizado, com planejamento e organização; Desenvolver parcerias e trabalhar de modo conjunto, em equipe e intersetorialmente; Buscar conhecimento e autodesenvolvimento.
**SAÚDE E SEGURANÇA PARA O TRABALHADOR**	Farmacologia e clínica médica; Urgência e emergência; Comunicação e expressão e didática; Tecnologias de Informação e Comunicação; Legislação e auditoria; Prevenção e promoção de saúde com planejamento estratégico e gestão de riscos; Saúde do trabalhador (saúde mental, ergonomia, higiene e segurança do trabalho, doenças ocupacionais e vigilância em saúde).	Realizar/adaptar o planejamento e ter tomada de decisão; Liderar e ter autonomia; Buscar a qualidade no trabalho e o seu autodesenvolvimento; Prever e prover materiais e insumos; Desenvolver ações educativas; Ter capacidade argumentativa; Executar registros e comunicação escrita; Dominar as habilidades técnicas e assistenciais; Supervisionar as práticas de biossegurança.
**PESQUISA**	Epidemiologia (bioestatística) e vigilância em saúde; Métodos e técnicas de pesquisa; Auditoria; Enfermagem baseada em evidências; Comunicação e expressão; Língua inglesa e informática.	Coletar, tratar e analisar dados; Buscar por conhecimento/estudar para oferecer/possuir respaldo técnico-científico; Definir os indicadores de saúde; Dominar a comunicação escrita, sistemas de registros de informações e gráfica; Ter planejamento e tomada de decisão, organização e gerir o tempo; Desenvolver parcerias.

## DISCUSSÃO

A análise dos dados possibilitou identificar as competências essenciais do ET conforme a sua práxis profissional e propor uma matriz de competências, considerada pela literatura como essencial às profissões^(24,25)^, em que a organização dos conhecimentos e habilidades sob a forma de uma matriz de competências permite a identificação das competências mínimas que se fazem necessárias e, ao mesmo tempo, caracterizam a atividade profissional^(26)^.

O modelo adotado como referencial teórico para análise e construção da proposta de matriz de competências elenca seis competências essenciais cujas habilidades se apresentam em níveis crescentes de complexidade, conforme nível de progressão na carreira^(16)^, o que não se aplica à realidade vivida pelo ET na indústria brasileira.

Destaca-se, entre as competências identificadas, quatro com caráter de transversalidade, denominadas jurídica, comunicação eficaz, relacional, e gerência da equipe e do serviço. As competências transversais apresentam conhecimentos e habilidades que perpassam as demais competências, e têm características de: multifuncionalidade (necessárias em diferentes facetas da vida); transferibilidade (usadas no mundo do trabalho, na vida pessoal e em sociedade, no desenvolvimento da carreira); são baseadas na cognição (constroem-se com reflexões individuais ativas, pelo pensamento crítico); multidimensionalidade (composta por diferentes *clusters* de competências, como cognitivo, interpessoal, empresarial, preparação para o trabalho); são aprendidas (processo contínuo por meio de diferentes contextos) e abrangentes (são amplos, envolvem explanações e a aplicação do conhecimento)^(27)^.

O desenvolvimento e a aplicabilidade das competências transversais no trabalho dependem da capacidade de articulação dos conhecimentos em resposta às mudanças de nível organizacional, setorial e institucional, integrando conteúdos (teoria e prática), o que exige seu aprimoramento diante das alterações de contexto laboral^(28)^.

Ao tratar sobre a competência jurídica, pesquisadores afirmam que, por se relacionar à ética e à legislação, ela exige o conhecimento, a aplicação e o respeito às leis que ordenam e orientam qualquer tipo ou modalidade de trabalho realizado ou não por esse profissional^(29)^, o que reforça as suas características de essencialidade e transversalidade^(30)^, comprovado ao identificar o seu núcleo de conhecimentos em cinco das competências relatadas, demonstrando que essa competência é fundamental na garantia de que as práticas do ET estejam alinhadas com as exigências legais e éticas, ao agir para propiciar um ambiente de trabalho seguro e saudável para todos os trabalhadores.

Para a competência gerência da equipe e do serviço, há estudos que destacam a necessidade de conhecimento nas áreas da estatística, Sistematização da Assistência de Enfermagem em Saúde Ocupacional, informática, planejamento estratégico, legislação (sanitária, trabalhista, de seguridade social e previdenciária), gestão de pessoas e trabalho em equipe^(8,9,31)^, corroborando os resultados identificados no estudo.

Destaca-se que o gerenciamento é uma das atividades privativas do enfermeiro, independentemente de seu contexto laboral. A importância da gestão em serviço agrega a implementação de modelos gerenciais que permitam planejar, decidir, organizar e controlar a prestação da assistência, por meio da utilização de práticas gerenciais voltadas a maior eficácia e eficiência na aplicação dos recursos^(32)^, o que, para o ET, torna-se um desafio cotidiano, na busca por melhores condições de trabalho e de qualidade dos serviços oferecidos aos colaboradores de uma organização. Assim, sua ação deve estar fundamentada em instrumentos, meios e competências.

Já a comunicação é competência essencial que se intersecciona com a gestão, identificada em ações junto à tomada de decisão, no emprego de habilidades sociais, visível no gerenciamento da equipe, no partilhar de informações atualizadas junto à gestão nas áreas estratégica e financeira^(33)^. Ao utilizar a comunicação, o ET estabelece um relacionamento interpessoal com a equipe, fato relevante para o desempenho dos profissionais e para o sucesso da organização. Tem-se, portanto, a pertinência com os conhecimentos e habilidades relacionados à competência relacional, e que se intersecciona com a comunicação eficaz.

No grupo das demais competências essenciais, os participantes destacaram a necessidade de domínio de mais de um idioma, seja o inglês ou o francês, por algumas empresas serem multinacionais e os trabalhadores precisarem se comunicar com empregadores e trabalhadores que não falam o português. A capacidade dos enfermeiros para interagir produtivamente com pessoas de origens culturais diferentes é necessária para propiciar maior qualidade e resultados dos serviços^(34,35)^, além de possibilitar conhecer outras estratégias e realidades de trabalho.

A reflexão sobre o aprimoramento do processo de trabalho como competência do ET se traduz na implementação de práticas que aumentam a eficiência, a segurança e a qualidade dos serviços de saúde ocupacional. O ET deve ser capaz de identificar áreas de melhoria, desenvolver estratégias eficazes e liderar iniciativas de mudança que beneficiem tanto os trabalhadores quanto a organização^(35,36)^.

Sabe-se que o ET é o responsável por identificar e avaliar possíveis riscos do ambiente de trabalho. Logo, a ele é requerida a implementação de medidas de prevenção e controle, além de orientar os colaboradores sobre as normas de segurança. Assim, ter a competência para a saúde e segurança no trabalho é relevante no cotidiano do seu trabalho, fato corroborado pela literatura científica^(9,37)^.

A competência pesquisa tem vários conhecimentos e habilidades que foram identificados em outras competências, o que reforça sua importância e aplicabilidade^(38,39)^.

Todas as competências demandam conhecimentos e habilidades específicas, mas é relevante expor que esses conhecimentos se entrelaçam nas diversas competências apresentadas. Além disso, tem-se habilidades recorrentes, como o relacionamento interpessoal, que se reflete em desenvolver parcerias e trabalho colaborativo, trabalhar em equipe, realizar *networking*. Essa habilidade é descrita como fundamental no exercício profissional em decorrência da interação com a equipe multiprofissional^(34)^.

Nessa direção, para que essas competências sejam incorporadas na práxis, estratégias podem ser implementadas pela organização ou pelo próprio trabalhador. Aos gestores, cabe incentivar o aprendizado contínuo para o desenvolvimento dos seus colaboradores, extrapolando modelos tradicionais que se direcionam a suprir lacunas de conhecimento ou habilidades pontuais no exercício profissional^(40)^.

Novas pesquisas devem ser conduzidas sobre as competências profissionais para outras categorias da área da saúde do trabalhador, a exemplo do técnico de enfermagem do trabalho.

### Limitações do estudo

Este estudo apresenta como limitações de acesso ao profissional a sua distribuição pulverizada no Brasil e a pandemia de SARS-CoV-2, ampliando as atividades do ET nas empresas (assistencial e gerencial), o que restringiu sua disponibilidade em participar do estudo.

### Contribuições para as áreas da enfermagem, saúde ou políticas públicas

A construção da uma matriz de competências para o ET deve orientar gestores e provocar a reflexão dos centros formadores nacionais na reorganização de seus projetos pedagógicos, adequando as disciplinas à práxis trabalhadora do ET, pelo aprimoramento dos conhecimentos e habilidades desses profissionais e área acadêmica, visando à mudança de perfil, ao reconhecimento profissional e à valorização. Cientificamente, espera-se o reconhecimento do fazer do ET, ao desvelar sua realidade profissional, no despontar de estudos nesta realidade, considerando a visão, a experiência e a construção da enfermagem do trabalho conforme seus atores.

## CONSIDERAÇÕES FINAIS

Identificaram-se, pela percepção do ET, as competências essenciais à sua práxis profissional, e baseado nelas, construiu-se uma matriz de competências profissionais. Esta matriz pode direcionar, de modo assertivo, a formação dos ETs conforme as necessidades existentes ao cargo e suas funções, ao reconhecer suas competências e identificar as lacunas existentes na formação desse profissional.

A matriz possibilita uma melhor visualização dos conhecimentos e habilidades de cada competência, o que deve favorecer o seu reconhecimento junto às organizações, com a cisão de paradigmas quanto ao seu inestimável valor à saúde do trabalhador, elevando a profissão a um novo patamar.

É premente investir em pesquisas que investiguem a práxis do ET no Brasil, auxiliando órgãos e sistemas de atenção à saúde no investimento em saúde do trabalhador e fortalecimento da área que trata da manutenção da saúde da população economicamente ativa no país.

## Data Availability

Os dados de pesquisa só estão disponíveis mediante solicitação.
